# Modified Proofreading PCR for Detection of Point Mutations, Insertions and Deletions Using a ddNTP-Blocked Primer

**DOI:** 10.1371/journal.pone.0123468

**Published:** 2015-04-27

**Authors:** Weiming Hao, Lujuan Fan, Qianqian Chen, Xiaoxiang Chen, Sichao Zhang, Ke Lan, Jian Lu, Chiyu Zhang

**Affiliations:** 1 Institute of Life Sciences, Jiangsu University, Zhenjiang, Jiangsu, China; 2 Pathogen Diagnostic Center, Institut Pasteur of Shanghai, Chinese Academy of Sciences, Shanghai, China; 3 Department of Gynecologic Oncology, Jiangsu Cancer Hospital, Affiliated Cancer Hospital of Nanjing Medical University, Nanjing, Jiangsu, China; 4 Huzhou Center for Disease Control and Prevention, Huzhou, Zhejiang, China; 5 School of Medicine, Jiangsu University, Zhenjiang, Jiangsu, China; Austrian Federal Research Centre for Forests BFW, AUSTRIA

## Abstract

The development of simple, accurate, rapid and cost-effective technologies for mutation detection is crucial to the early diagnosis and prevention of numerous genetic diseases, pharmacogenetics, and drug resistance. Proofreading PCR (PR-PCR) was developed for mutation detection in 1998 but is rarely applied due to its low efficiency in allele discrimination. Here we developed a modified PR-PCR method using a ddNTP-blocked primer and a mixture of DNA polymerases with and without the 3'-5' proofreading function. The ddNTP-blocked primer exhibited the best blocking efficiency to avoid nonspecific primer extension while the mixture of a tiny amount of high-fidelity DNA polymerase with a routine amount of Taq DNA polymerase provided the best discrimination and amplification effects. The modified PR-PCR method is quite capable of detecting various mutation types, including point mutations and insertions/deletions (indels), and allows discrimination amplification when the mismatch is located within the last eight nucleotides from the 3'-end of the ddNTP-blocked primer. The modified PR-PCR has a sensitivity of 1-5 × 10^2^ copies and a selectivity of 5 × 10^-5^ mutant among 10^7^ copies of wild-type DNA. It showed a 100% accuracy rate in the detection of P72R germ-line mutation in the *TP53* gene among 60 clinical blood samples, and a high potential to detect rifampin-resistant mutations at low frequency in *Mycobacterium tuberculosis* using an adaptor and a fusion-blocked primer. These results suggest that the modified PR-PCR technique is effective in detection of various mutations or polymorphisms as a simple, sensitive and promising approach.

## Introduction

Human diseases such as cancer, cardiovascular disease and autoimmune diseases are always associated not only with environmental factors, but also with genetic factors. The most commonly observed genetic variations are germ-line and somatic mutations (including point mutations, insertions and deletions), many of which are deleterious and affect or change the functions and/or expression of certain proteins if they occur in coding and/or non-coding (i.e. promoter) regions [1–3]. In addition, some point mutations have been found to be associated with human responses to anti-cancer targeted therapy and the development of drug resistance of pathogens [4]. Therefore, accurate and rapid detection of these mutations plays an increasingly important role in the early diagnosis and prognosis of various genetic diseases, and discovery of drug resistance [1–4].

To date, a wide variety of methods have been developed to detect genetic variants, especially point mutations in genes, which can be mainly classified into two categories: allele discrimination strategies and sequencing methods [5]. The allele discrimination strategies are usually based on allele-specific reactions including primer extension, hybridization [5], ligase-mediated amplification reaction (LDR) [6,7], enzymatic cleavage [8–10] and other methods (e.g. single-strand conformation polymorphism analysis (SSCP) [11,12], pyrophosphorolysis-activated polymerization (PAP) [13–15] and high resolution melting analysis (HRM)) (S1 Table) [16,17]. The sequencing methods, including conventional Sanger sequencing [18–20] and next-generation sequencing (NGS) [20,21], are often used as the gold standard for variant confirmation (S1 Table) [5,19,20]. However, some allele discrimination methods are laborious, time-consuming and requiring expensive instruments [20,22], or present poor accuracy and sensitivity in the detection of variations, especially those at lower frequencies [12,19,20], which severely limit their wide applications. Typically, allele-specific PCR (AS-PCR) is well known as a simple, fast and cost-effective method in mutation detection and polymorphism genotyping, but the biggest limitation is that the allele-specific primer can often be non-specifically extended by most DNA polymerases, even if there is a mismatch with the template at its 3'-end, which results in false positive results (S1A Fig) [3,22,23].

As a new kind of allele discrimination strategy, proofreading PCR (PR-PCR) was first developed for mutation detection in 1998 [24]. This technique exploits the 3'-5' exonuclease (proofreading) activity of high-fidelity DNA polymerase to remove the blocked 3'-terminal nucleotide from the inert primer when the blocked nucleotide mismatches with the template and then to extend the primer with a free 3'-hydroxyl group (-OH). If the inert primer with a blocked 3'-OH matches with the template completely, it will not be extended since the blocked 3'-terminal nucleotide is unable to be removed. However, this simple and cost-effective approach has still not been widely applied due to its low efficiency and sensitivity in allele discrimination, which are also the common problems of some allele-specific strategies (S1 Table). Here we report a novel modified PR-PCR method that uses a dideoxynucleotide(ddNTP)-blocked primer and a mixture of DNA polymerases with and without the 3'-5' proofreading function. The new method exhibits better performance in allele discrimination and higher sensitivity in comparison with the conventional PR-PCR and can be used to rapidly and accurately detect various variations, especially those at lower frequency.

## Materials and Methods

### Ethics statement

This study was performed according to the Helsinki II Declaration and was approved by the medical ethics committee of Jiangsu Cancer Hospital. Sixty clinical blood samples were obtained from breast cancer patients at Jiangsu Cancer Hospital. Written informed consent was obtained from all subjects.

### Primer design and synthesis

All primers were designed using the Primer Premier 5.0 program. For point mutation detection, an allele-specific primer matching the wild-type sequence and a common primer matching both wild-type and mutant sequences were designed. In addition, three blocked primers were synthesized by blocking the 3'-terminal nucleotide with 3'-Pi or-Amino C6(-NH_2_), or replacing it with ddCTP. The blocked primers have same sequences as the allele-specific primer. All primers were synthesized commercially by BioSune Biotechnology Co., Ltd. (Shanghai, China).

### Construction of various mutants

The wild-type template was obtained by constructing a recombinant pMD18-T plasmid (TaKaRa, Dalian China) containing a fragment of human genomic DNA. To evaluate the applicability of the proofreading PCR method, a series of mutant plasmids containing single-base mutations (including point mutations, deletions and insertions) at different positions were constructed using site-directed mutagenesis technology. All plasmids containing the wild-type fragment and various mutations were used as the templates after purification and confirmation via direct sequencing.

### Detection of various mutants

Both the wild-type and mutant templates (plasmids) were amplified using an allele-specific 3'-ddC-blocked forward primer and an unblocked reverse primer. The PCR amplifications were performed in a total volume of 20 μL with 2 μL of (10×) Taq PCR buffer, 0.2 mM deoxynucleotide-triphosphate (dNTPs), 1 ng of template, 0.15 units (U) of KOD FX DNA polymerase (TOYOBO, Osaka, Japan), 1 U of Taq DNA polymerase (TaKaRa, Dalian, China) and 0.3 μM each of reverse primer and 3'-ddC-blocked forward primer. The reactions were carried out under cycling conditions of pre-denaturation at 94°C for 2 min, followed by 30 cycles of denaturation at 94°C for 20 s, annealing at 62.2°C for 20 s and extension at 72°C for 25 s (the annealing temperature used for the amplification of Mu*8 was 62.9°C). The PCR products were visualized via 1.5% agarose gel electrophoresis.

### Confirmation test using human cancer cell lines and clinical samples

To further evaluate the detection efficiency of the modified PR-PCR in realistic samples, two breast cancer cell lines HCC1937(ATCC#CRL-2336) and MCF-7(ATCC#HTB-22), purchased from the Shanghai Institutes for Biological Sciences, Chinese Academy of Sciences (Shanghai, China) and 60 clinical blood samples obtained from breast cancer patients were prepared. HCC1937 that carries a homozygous *TP53* mutation in codon 306 (p.R306X, c.916C>T) was used as the mutant sample, while MCF-7 that does not carry this mutation was used as the wild-type sample [25]. Both cell lines were cultured following ATCC protocols.

Genomic DNA (gDNA) was extracted from both cell lines using the MiniBEST Universal Genomic DNA Extraction Kit (TaKaRa, Dalian, China) following the manufacturer’s instructions. For the 60 blood samples, gDNA was extracted using the TIANamp Blood DNA Kit (TIANGEN, Beijing, China) according to the manufacturer’s instructions. The extracted gDNA was eluted into 70 μL of (1×) TE buffer (pH 8.0) and then quantified using a Nanodrop-1000 Spectrophotometer (Thermo Fisher Scientific, Wilmington, DE).

For the clinical samples, the modified PR-PCR was performed in a total volume of 25 μL reaction mixture including 20 ng of gDNA, 3.75 μL of (5×) PrimeSTAR buffer, 1.25 μL of (5×) Taq PCR buffer, 0.2 mM dNTPs, 0.2 U of PrimeSTAR HS DNA polymerase, 0.7 U of Taq DNA polymerase, 1 μL of DMSO and 0.3 μM each of reverse primer, fusion-blocked forward primer and adaptor. The reactions were carried out under cycling conditions of pre-denaturation at 94°C for 2 min; 40 cycles of denaturation at 98°C for 10 s, annealing at 59°C for 20 s and extension at 72°C for 18 s. The PCR products were visualized via 1.5% agarose gel electrophoresis.

To confirm the results obtained from the modified PR-PCR, a 430 bp fragment of the *TP53* gene containing the P72R mutation was amplified via routine PCR (S2 Table). The PCR products were purified and sent to BioSune Biotechnology Co., Ltd. for sequencing.

### Detection of rifampin-resistant mutations in *Mycobacterium tuberculosis (TB)*

Three rifampin-resistant (S479, S643, and S748) and three rifampin-sensitive (N15, N7, and N9) TB strains were isolated from the Huzhou Center for Disease Control and Prevention. Rifampin resistance and susceptibility of TB strains was determined using the proportional method on Löwenstein-Jensen medium with serial dilutions of rifampin, in accordance with the Deutsches Institut für Normung (DIN) guidelines (DIN 58943–8).

Two most common rifampin-resistant mutations, H526Y (**C**AC>**T**AC) and S531L (T**C**G>T**T**G) [26] in the *rpoB* gene of TB were detected using the modified PR-PCR. All reactions were performed in a total volume of 20 μL containing a mixture of 2 μL of (10×) Taq PCR buffer, 0.2 mM dNTPs, 1 U of Taq DNA polymerase, 0.15 U of PrimeSTAR HS DNA polymerase, 5 ng of gDNA (equal to approximately 1×10^6^ copies of the TB genome) and 0.3 μM each of 3'-blocked forward primer and common reverse primer. The PCR cycling conditions consisted of pre-denaturation at 95°C for 1 min, followed by 35 cycles of denaturation at 95°C for 15 s, annealing at 59°C for 15 s and extension at 72°C for 15 s.

To confirm the results obtained from the modified PR-PCR, a 493 bp fragment of the *rpoB* gene encompassing the two mutation sites from six TB strains was sequenced using the 454 GS-FLX system (Roche Diagnostics Corporation). Approximately 1×10^9^ molecules of the final normalized, adaptor and sequencing key-ligated 493 bp amplicon were prepared and sequenced according to the published Roche 454 GS FLX Titanium protocols.

## Results and Discussion

### Blocking efficiency of various kinds of blocking primers in PCR amplifications mediated via Taq DNA polymerase and high-fidelity DNA polymerase respectively

**Fig 1:**
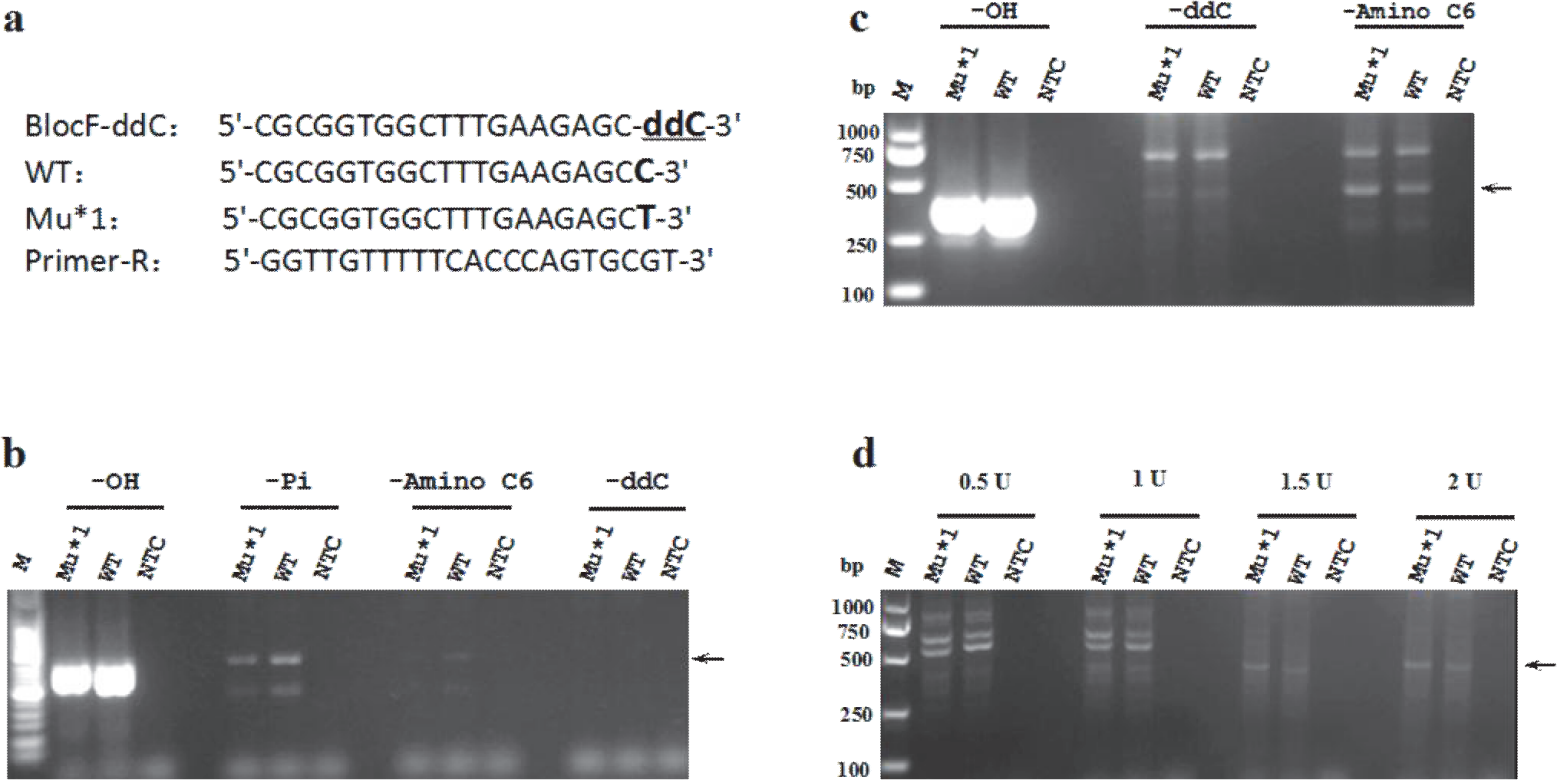
Blocking efficiency of three kinds of blocked primers in PCR amplifications mediated by Taq DNA polymerase and high-fidelity DNA polymerase. (a) Primer and template sets. WT and Mu indicate wild-type and mutant types, respectively. The 3'-blocked forward primer completely matches the WT sequence but forms a mismatch with the Mu sequence (Mu*1) at the 3'-end. The reverse primer matches both the WT and Mu sequences. The 3'-terminal nucleotide of the blocked primer is blocked with-Pi or-Amino C6, or replaced with ddCTP. (b) Blocking efficiency of-Pi,-Amino C6 or-ddC in PCR mediated by Taq DNA polymerase. (c) Blocking efficiency of-Pi,-Amino C6 or-ddC in PCR mediated by high-fidelity DNA polymerase (KOD FX). (d) Discrimination efficiency of the typical proofreading-PCR medicated by various amounts (0.5, 1, 1.5 and 2 U) of KOD FX DNA polymerase using the ddC-blocked primer. All PCR amplifications were performed in a total volume of 20 μL containing 1 U of Taq or KOD FX DNA polymerase (Panel b and c) or various amounts of KOD FX DNA polymerase (Panel d). The cycling conditions consisted of pre-denaturation at 94°C for 2 min, followed by 30 cycles of denaturation at 94°C for 20 s, annealing at 56°C for 20 s and extension at 72°C for 25 s. NTC: no-template control.

The blocking efficiency of inert primers is one of the most important factors affecting the specificity and accuracy of PR-PCR. In the previous studies, two kinds of chemical groups,-Pi and-Amino C6 (-NH_2_), were used to block the 3'-OH of the primer [24,27,28]. Here we designed a new kind of inert primer, a ddNTP-blocked primer, which contains a ddNTP at the 3'-end of the allele-specific primer. The ddNTP-blocked primer can not be extended by DNA polymerase because of the lack of free 3'-OH. We first performed AS-PCR assays with Taq DNA polymerase to test and compare the blocking efficiencies of the three kinds of inert primers mentioned above. A wild-type template that completely matched the allele-specific blocked primer and a mutant template that contained a C-T mismatch with the 3'-end of the allele-specific primer were used in the reactions (Fig 1A). The results showed that the extension of the-NH_2_- and ddNTP-modified primers could be well inhibited no matter which type of template (wild or mutant type) was used, whereas the extension of the-Pi-modified primer was not completely inhibited (Fig 1B).

We further performed the routine PR-PCR assay using high-fidelity DNA polymerase instead of Taq DNA polymerase. Only the extension of the ddNTP-blocked primer could be inhibited effectively no matter which type of template was used. For the-NH_2_-modified primer, the PR-PCR showed not only low discrimination between the wild-type and the mutant templates, but also low amplification efficiency (Fig 1C). To test whether increasing the amount of high-fidelity DNA polymerase would abolish the blocking efficiency of ddNTP-blocked primer, we further performed the reactions using 0.5, 1, 1.5 and 2 U of high-fidelity DNA polymerase, respectively (Fig 1D). The results showed that increasing the amount of high-fidelity DNA polymerase to 1.5 and 2 U per reaction slightly increased the amount of specific PCR products no matter which type of template was used (Fig 1D and S1B Fig), indicating that the increase in the amount of high-fidelity DNA polymerase slightly enhances amplification of specific PCR products, but does not substantially improve discrimination efficiency between the wild-type and the mutant templates. These results suggest that the routine PR-PCR is unsuitable for accurate detection of point mutations since it can result in false positive results.

### Development of a modified PR-PCR method using the dideoxynucleotide-blocked primer and a mixture of Taq DNA polymerase and high-fidelity DNA polymerase

**Fig 2:**
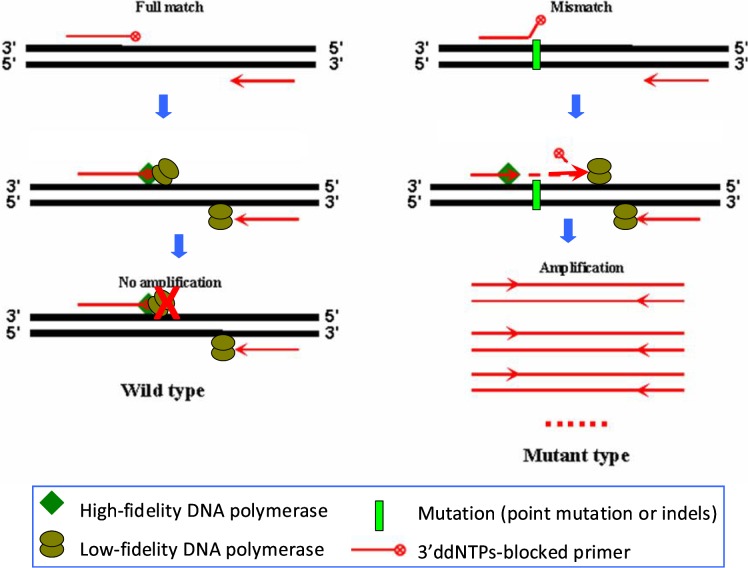
Principle of the modified proofreading PCR (PR-PCR) method for mutation detection. The high-fidelity and low-fidelity DNA polymerases correspond to the enzymes with and without 3', 5'-exonuclease activity, respectively.

The experimental results above indicate that two major factors affect the efficiency of the PR-PCR in mutation detection. One is that the modification of the 3'-end of a primer with-NH_2_ or-Pi is unable to completely block primer extension (Fig 1C). The other is that the PR-PCR using only high-fidelity DNA polymerase exhibits low amplification efficiency and allows a small quantity of primer to be extended via non-specific removal of the 3'-blocked nucleotide even if the blocked primer completely matches the template (Fig 1D). To solve these problems, we developed a modified PR-PCR strategy (Fig 2). In the new strategy, we selected the ddNTP-blocked primer, which appears to have the best blocking effect (Fig 1B and 1C), as the allele-specific primer to improve the specificity of PR-PCR. In addition, we largely decreased the amount of high-fidelity DNA polymerase to avoid the non-specific primer extension and added a routine amount of Taq DNA polymerase into the reaction to improve amplification efficiency. The major role of the tiny amount of high-fidelity DNA polymerase in the reaction is to activate primer extension by specifically removing the 3'-blocked nucleotide under the presence of a mismatch between the primer and the template (Fig 2).

**Fig 3:**
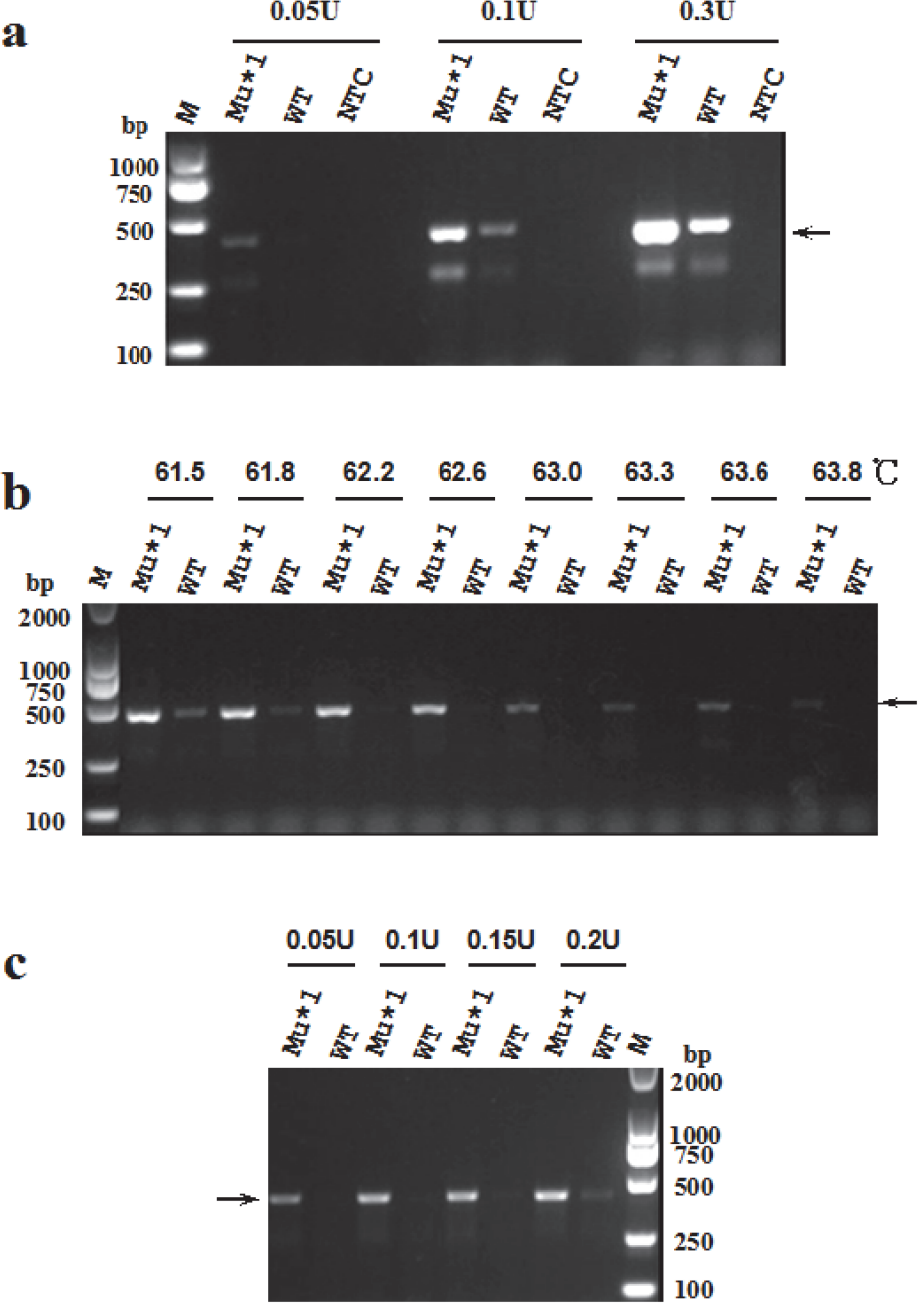
Optimization of annealing temperature and amount of high-fidelity DNA polymerase in the modified PR-PCR. (a) Discrimination efficiency of the modified PR-PCR for known mutations using a mixture of 1 U of Taq DNA polymerase and various amount (0.05, 0.1 and 0.3 U) of KOD FX DNA polymerase in the reaction. The PCR amplifications were performed in a total volume of 20 μL under cycling conditions consisting of pre-denaturation at 94°C for 2 min, followed by 30 cycles of denaturation at 94°C for 20 s, annealing at 56°C for 20 s and extension at 72°C for 25 s. (b) Optimization of annealing temperature for the modified PR-PCR using a mixture of 1 U of Taq DNA polymerase and 0.1 U of KOD FX DNA polymerase. As the optimal discrimination efficiency was obtained when the annealing temperature was within a scope of 61.8°C–62.6°C, the results from 50°C to 61.2°C were not shown. The temperature 62.2°C was selected for subsequent experiments. (c) Optimization of amount of high-fidelity DNA polymerase for the modified PR-PCR with an input of 1 U of Taq DNA polymerase at an annealing temperature of 62.2°C.

To determine the appropriate amount of high-fidelity DNA polymerase for the modified PR-PCR, we performed a preliminary experiment using a combination of 1 U of Taq DNA polymerase together with 0.05, 0.1 or 0.3 U of KOD FX DNA polymerase at an annealing temperature of 56°C in a 20 μL reaction (Fig 3A). The result showed that when the amount of high-fidelity DNA polymerase was decreased to 0.05 U, only the mutant sequence could be slightly amplified (Fig 3A), indicating that the decrease in the amount of high-fidelity DNA polymerase indeed inhibited the non-specific amplification of the wild-type sequence and enhanced the discrimination efficiency.

The formation of a mismatch between primer and template is a prerequisite for the activation of the 3'-5' exonuclease activity of high-fidelity DNA polymerase to remove the 3'-mismatched nucleotide of the primer. A relatively high temperature (close to or above the melting temperature of the primer) will facilitate the instability of DNA duplex, and enhance the dissociation of mismatched bases between primer and template, which may favor the recognition of mismatched bases by high-fidelity DNA polymerase. Therefore, slightly increasing the annealing temperature is believed to be able to improve the efficiency of high-fidelity DNA polymerase to remove the 3'-mismatched nucleotide of the primer, and accordingly improve the performance of the modified PR-PCR method.

The melting temperatures of the blocked primer and the normal reverse primer used in the assay were 61.6°C and 55.7°C, respectively. To test this hypothesis and to optimize the annealing temperature for the modified PR-PCR, we performed preliminary assays at ten different annealing temperatures from 55.1°C to 69.6°C. As expected, only at 63.2°C, a temperature higher than the melting temperatures of the primers, the mutant template could be specifically amplified, but the wild-type template could not (data not shown). When the annealing temperature was below 63.2°C (i.e. 55.1°C-61.2°C), both the wild-type and the mutant templates were amplified, and when the temperature was above 63.2°C (i.e. 65.2°C-69.6°C), both the wild-type and the mutant templates failed to be amplified (data not shown). So, we further optimized the annealing temperature for the modified PR-PCR at a range of 61.5°C-63.8°C. The optimal allele discrimination efficiency was observed at the annealing temperatures of 61.5°C-62.6°C, higher than that often used in the routine PCR (about 55°C) (Fig 3B). Then we selected 62.2°C as the annealing temperature of the modified PR-PCR to further optimize the amount of high-fidelity DNA polymerase. The results showed that the use of 0.1–0.15 U of high-fidelity DNA polymerase combined with 1 U of Taq DNA polymerase in per 20 μL reaction resulted in the best discrimination and amplification effects (Fig 3C). Thus, a mix of 0.1–0.15 U of high-fidelity DNA polymerase with 1 U of Taq DNA polymerase in per 20 μL reaction was selected for the subsequent experiments.

### Broad applicability of the modified PR-PCR in detection of various mutation types

It is well known that high-fidelity DNA polymerase has a proof reading function through removing mismatched nucleotides in a newly synthesized DNA strand. Mismatch that can activate the 3'-5' exonuclease activity of the high-fidelity DNA polymerase is not always limited to be the last nucleotide of the newly synthesized DNA strand or primer [29,30]. Therefore, mismatch near the 3'-end of the primer should also activate the 3'-5' exonuclease activity of the high-fidelity DNA polymerase and initiate the modified PR-PCR amplification. This implies that there is a large flexibility in design of an allele-specific primer for the modified PR-PCR (that is, the mismatched site can be designed at the position before the 3'-end of the primer). On the other hand, because indels always cause downstream frameshift mismatches, the indels between primer and template should also be able to initiate the modified PR-PCR amplification. These imply that the modified PR-PCR method can detect various mutation types including point mutation and indels.

**Fig 4:**
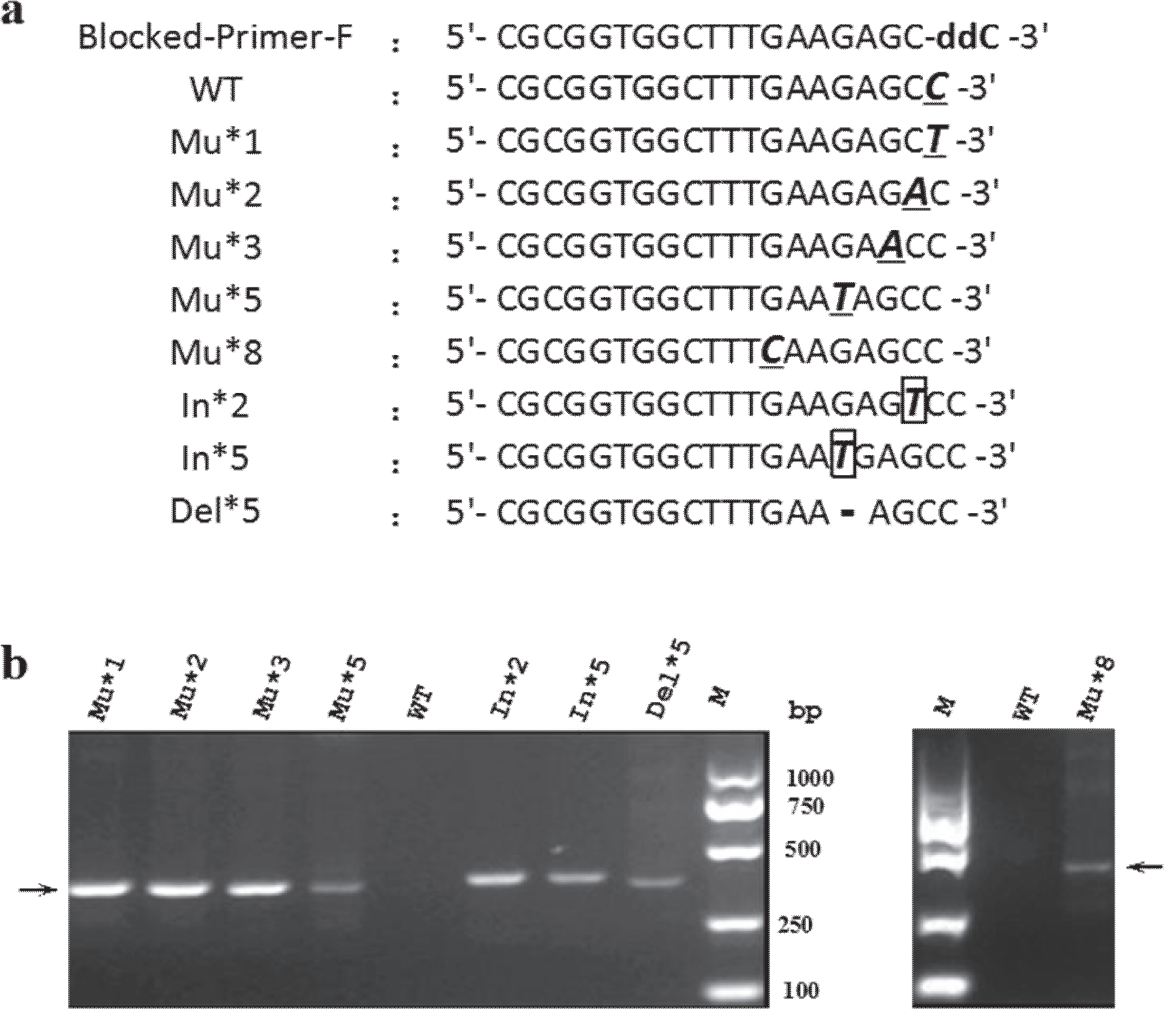
Detection of various mutation types using the modified PR-PCR. (a) Primer and template sets. WT and Mu indicate wild-type and mutant sequences, respectively. In and Del represent insertion and deletion mutations, respectively. For Mu*n (including In*n and Del*n), the **n** represents the distance from the mismatch to the 3'-end of the blocked primer. The point mutation, insertion and deletion sites are highlighted with underline, rectangle and gap (-), respectively. (b) Gel electrophoresis of the amplicons of various mutations obtained using the modified PR-PCR.

To demonstrate the applicability of the modified PR-PCR method in detection of various mutations, we constructed eight mutants, including five point mutants (Mu*1, Mu*2, Mu*3, Mu*5 and Mu*8), two insertion mutants (In*2 and In*5) and one deletion mutant (Del*5), which form a mismatch with the ddNTP-blocked primer at positions 1, 2, 3, 5, 8, 2, 5, and 5 from the 3'-end of the primer, respectively (Fig 4A). The amplification experiments of all these mutants except for Mu*8, together with the wild-type template, were performed under the optimized conditions described above. The amplification experiment of Mu*8 was performed together with the wild-type template under a different condition with 0.2 U of KOD FX DNA polymerase in 20 μL reaction and an annealing temperature of 62.9°C in cycle program. All eight mutants were effectively amplified by the modified PR-PCR, but the wild-type template that completely matched the blocked primer was not (Fig 4B). Additionally, the amplification efficiency appeared to be associated with the distance of the mismatch to the 3'-end of the blocked primer. The shorter the distance of the mismatch to the 3'-end of the primer was, the higher the amplification efficiency was (Fig 4B). According to the results above, the maximum distance that allowed discrimination amplification using the modified PR-PCR method was eight bases from the mismatch to the 3'-end of the ddNTP-blocked primer. These results indicate that the modified PR-PCR has broad applicability in detection of various mutation types.

### Selectivity and sensitivity of the modified PR-PCR

**Fig 5:**
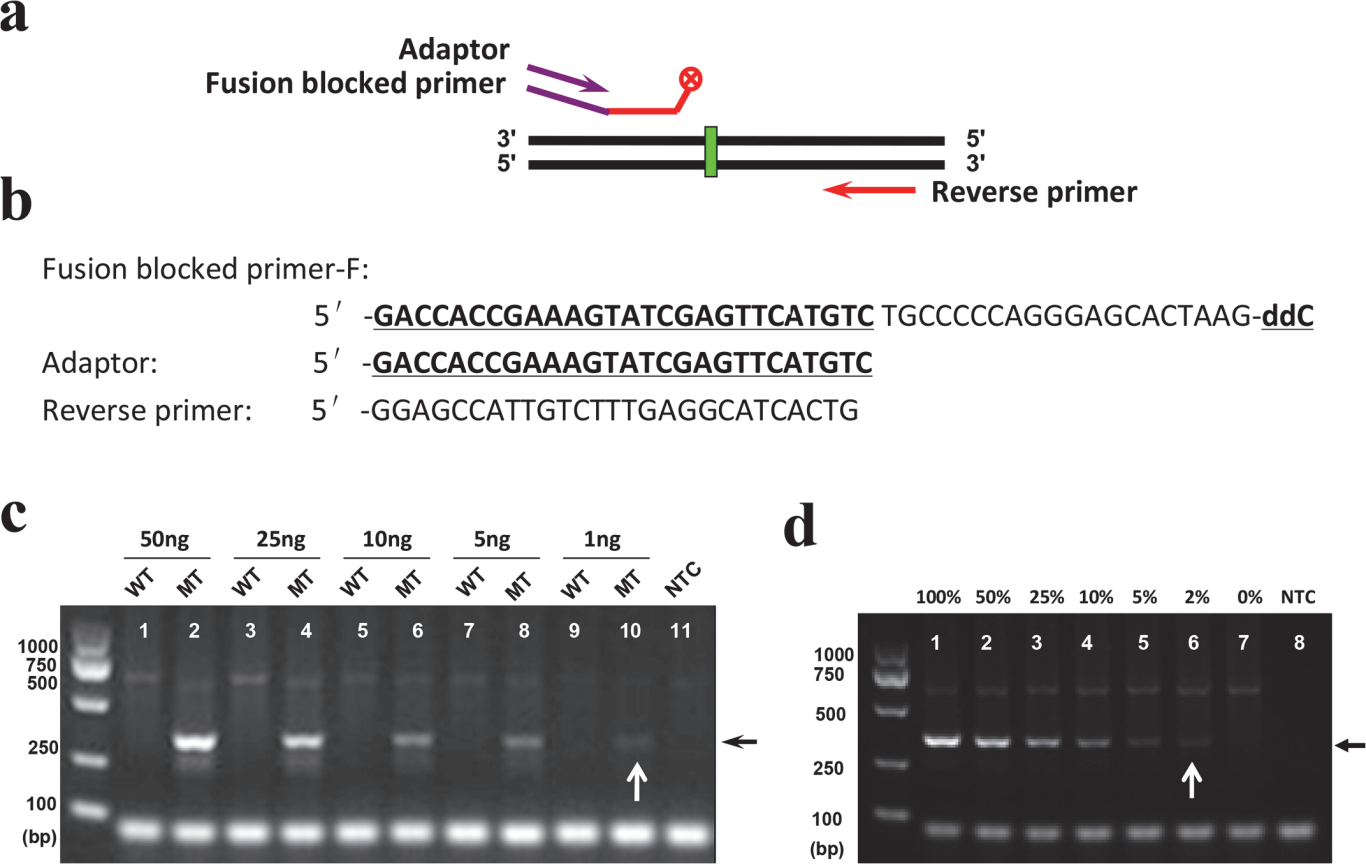
Sensitivity and selectivity of the modified PR-PCR for mutation detection using a fusion-blocked primer and adaptor. (a) Diagram of the fusion-blocked primer and adaptor used in the reactions. (b) Primer and adaptor sequences. (c) Sensitivity of the modified PR-PCR. (d) Selectivity of the modified PR-PCR. All reactions were performed in a total volume of 25 μL containing a mixture of 1 μL of gDNA template at 50 ng/μL, 2.5 μL of (10×) Taq PCR buffer, 0.2 mM dNTPs, 0.15 U of PrimeSTAR HS DNA polymerase, 0.75 U of Taq DNA polymerase, 1 μL of DMSO and 0.3 μM each of the reverse primer, fusion-blocked forward primer and adaptor. The DNA templates were prepared by mixing different amounts of MCF-7 (WT) gDNA (from 0 to 50 ng) among HCC1937 (mutant type, MT) gDNA (from 50 to 0 ng) with concentrations from 0 to 100%. The reactions were performed under cycling conditions consisting of pre-denaturation at 94°C for 2 min, followed by 40 cycles of denaturation at 98°C for 10 s, annealing at 59°C for 30 s and extension at 72°C for 20 s.

To evaluate the selectivity of the modified PR-PCR, we first used the recombinant plasmids at a concentration of 10^7^ copies per microliter as the templates to perform the experiments. The selectivity of the modified PR-PCR could reach a frequency of 1 × 10^−3^ to 5× 10^−3^ mutant alleles among wild-type DNA (S2A Fig). According to the principle of PR-PCR amplification, the blocked primer can be extended only when the 3'-ddNTP-blocked nucleotide of the primer is removed (Fig 2). Therefore, the removal of the 3'-ddNTP-blocked nucleotide of the primer by high-fidelity DNA polymerase is the rate-limiting step determining the efficiency of PR-PCR. Because increasing the amount of high-fidelity DNA polymerase did not improve the discrimination efficiency between the wild-type and mutant templates (Fig 1D), the effort to improve efficiency of PR-PCR should focus on the amplification process of newly synthesized products, rather than the step of removing the 3'-ddNTP-blocked nucleotide. In order to improve the amplification efficiency of the modified PR-PCR and obtain high detection sensitivity, we designed a fusion-blocked primer by adding an adaptor sequence to the 5'-end of the blocked primer and then performed same amplification reactions using both the fusion-blocked primer and the adaptor instead of the blocked primer alone (Fig 5A). Since the newly synthesized PCR products in each cycle can be used as templates of both the fusion-blocked/reverse primer pair and the adaptor/reverse primer pair in the next cycle, the amplification efficiency can be greatly improved. As expected, when the fusion-blocked primer and adaptor were used, the modified PR-PCR was able to detect a frequency of 5×10^−5^ mutant plasmids among wild-type plasmids (S2B and S2C Fig).

We then used the gDNA of two breast cell lines as templates to further evaluate the sensitivity and selectivity of the new method. The R306X germ-line mutation in the *TP53* gene was detected. The method was able to detect 10 ng of gDNA from HCC1937 (mutant type) cells (data not shown). When the fusion-blocked primer and adaptor were used (Fig 5B), the sensitivity of the method reached 1 ng of HCC1937 gDNA (equal to approximately 2.7 × 10^2^ copies) (Fig 5C). When a mixture of templates including gDNA from both cell lines was used, 2% of the R306X mutation could be detected with a 50 ng gDNA input (Fig 5D). These results indicate that the modified PR-PCR has high detection sensitivity and selectivity.

### Detection of the *TP53* P72R mutation among clinical samples using the modified PR-PCR

**Fig 6:**
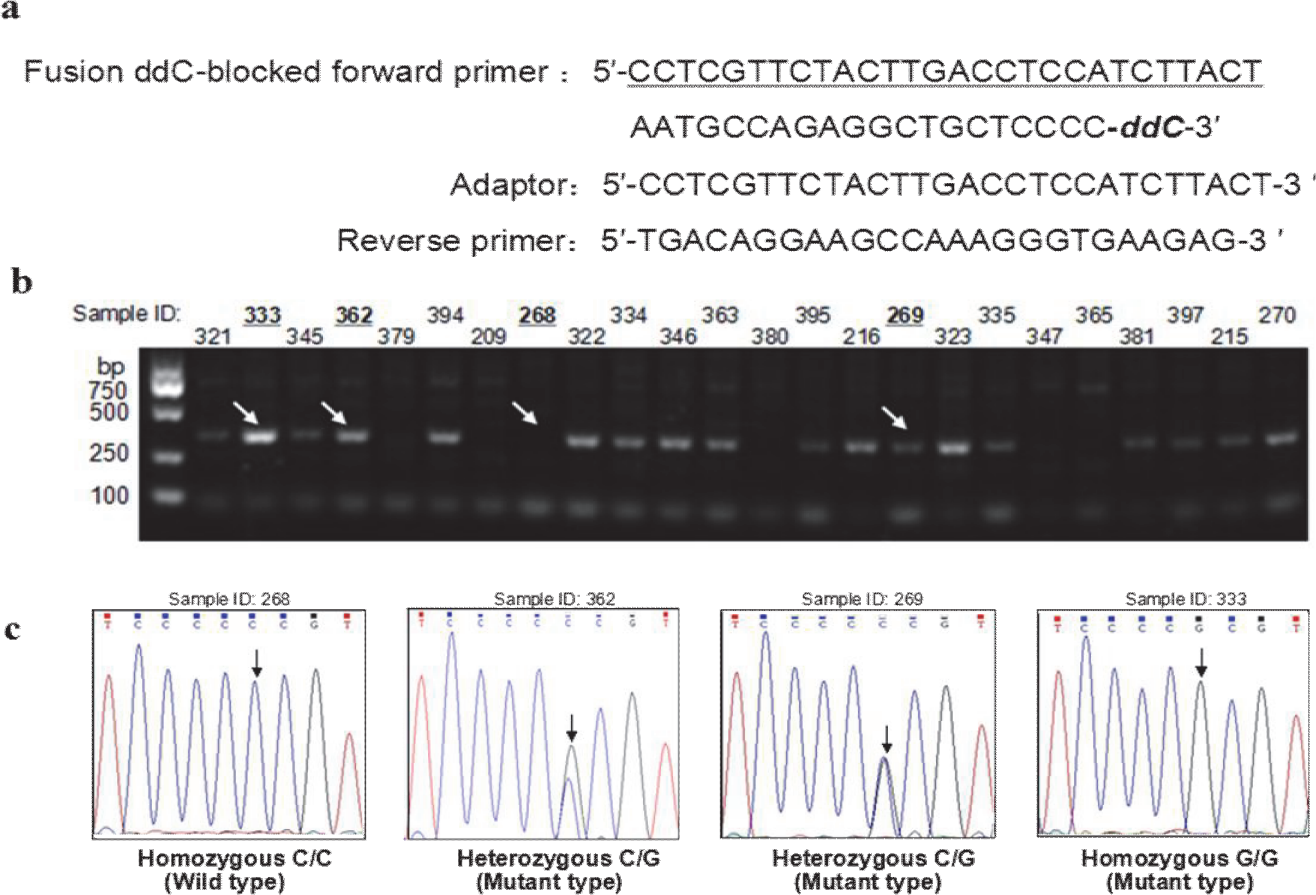
Detection of the P72R mutation in the *TP53* gene using the modified PR-PCR. (a) Primer and adaptor sequences. (b) P72R-specific amplification of 60 clinical samples using the modified PR-PCR. The arrows highlight the amplification products of four representatives of the 60 clinical samples with sequencing maps shown in Panel c. (c) Sequencing map of four representatives of the 60 clinical samples.

To test the applicability of the modified method to clinical samples, we collected 60 blood samples from breast cancer patients and used the modified method with the fusion-blocked primer and adaptor to detect the incidence of P72R (c.215C>G, **rs**1042522) germ-line mutation of the *TP53* gene among these samples (Fig 6A) since P72R was previously demonstrated to associate with breast cancer and other cancer types [31]. The specific PCR product (322 bp) was detected in 47 of the 60 samples (Fig 6B), suggesting that 78.3% of the subjects harbor the P72R mutation. To confirm these results, a fragment of the *TP53* gene encompassing this mutation was amplified and sequenced. The sequencing results showed that all the 47 positive samples identified via the modified PR-PCR method indeed carried the P72R mutation (including 33 heterozygous mutations and 14 homozygous mutations), while the other 13 negative samples were homozygous wild-type (Fig 6C). These indicate that the modified PR-PCR method can detect polymorphisms accurately.

### Detection of rifampin-resistant mutations from clinical isolates of *Mycobacterium tuberculosis* using the modified PR-PCR

**Fig 7:**
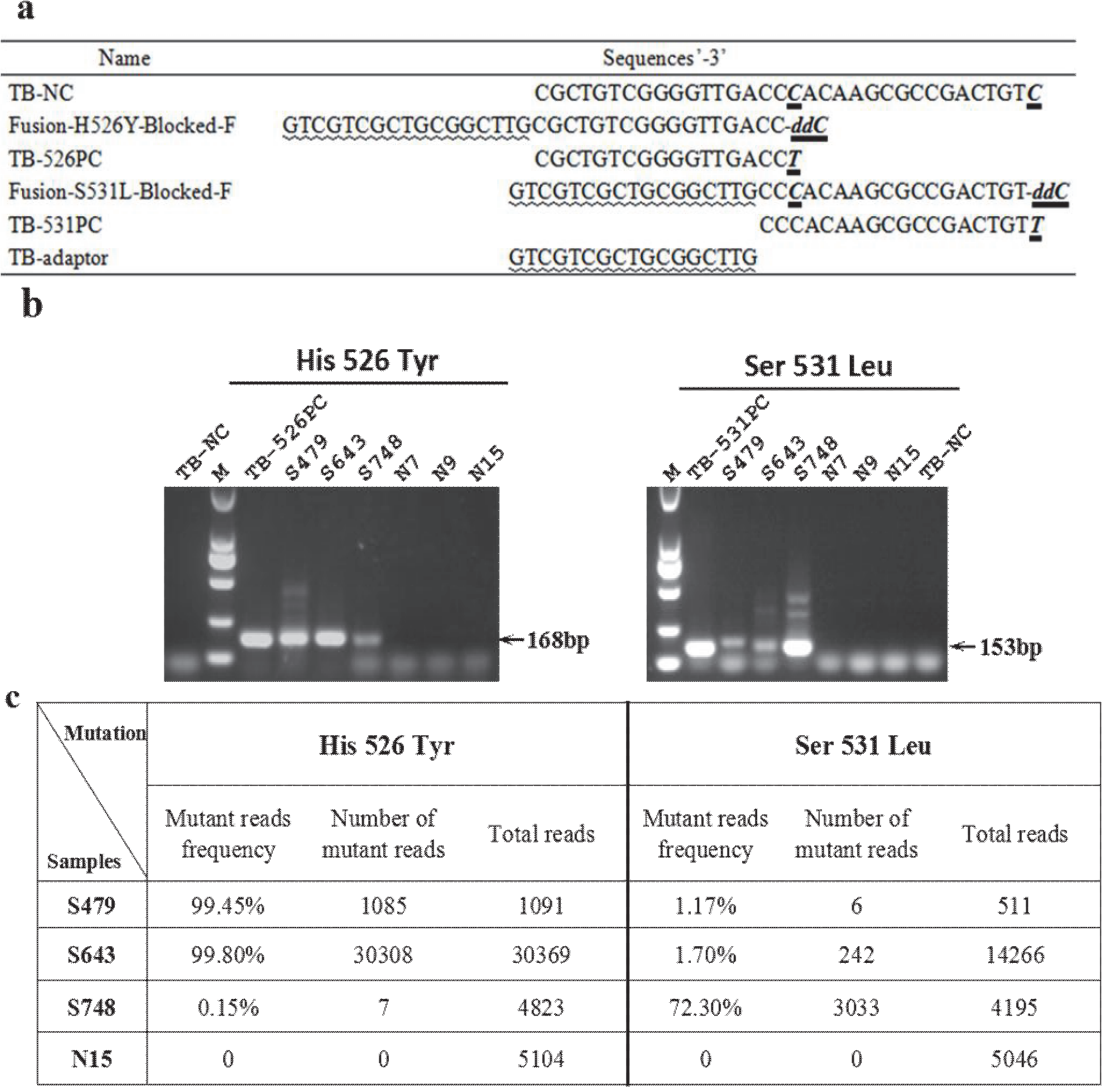
Detection of rifampin-resistant mutations in TB using the modified PR-PCR and next-generation sequencing. (a) Primer and template sequences. TB-NC is a negative control plasmid containing the *rpoB* gene fragment without any rifampin-sensitive mutations. TB-H526Y and TB-S531L are positive control plasmids containing the *rpoB* gene fragment with the H526Y(CAC→TAC) and S531L(TCG→TTG) mutations, respectively. (b) Gel electrophoresis of the amplicons for the two mutations in the TB *rpoB* gene using the modified PR-PCR. (c) Detection of two rifampin-resistant TB mutants using 454 high-throughput sequencing.

To further test the applicability of the modified method in analysis of drug resistance, we detected two of the most common mutations, H526Y (**C**AC>**T**AC) and S531L (T**C**G>T**T**G) in the *rpoB* gene of rifampin-resistant *Mycobacterium tuberculosis* (TB) strains [26]. The sequences of the wild-type TB, resistant TB and the primers are shown in Fig 7A. Three rifampin-resistant (S479, S643 and S748) and three rifampin-sensitive (N7, N9 and N15) strains were used in the assay. The specific PCR products for both mutations were only obtained from the three rifampin-resistant *TB* isolates (S479, S643 and S748), but not from the rifampin-sensitive TB isolates (N7, N9 and N15) (Fig 7B). In addition, the amounts of the H526Y-specific amplification product from isolate S748 and the S531L-specific amplification products from isolates S479 and S643 appeared to be less than the others, suggesting that the mutations may exist with a very low frequency. To confirm the results and evaluate the ability of the modified PR-PCR in detection of rare mutations, a 493 bp fragment of the *rpoB* gene encompassing the two mutation sites was amplified from the three rifampin-resistant TB isolates and one rifampin-sensitive TB isolate (N15) and subjected to 454 high-throughput sequencing. For H526Y and S531L, a total of 41387 and 24018 reads were obtained, respectively (Fig 7C). The frequencies of H526Y among the isolates S479, S643 and S748 were 99.5%, 99.8% and 0.15%, respectively. The frequencies of S531L among isolates S479, S643 and S748 were 1.2%, 1.7% and 72.3%, respectively. No mutant was found in the rifampin-sensitive isolate N15 for either H526Y or S531L (Fig 7C). The results of high-throughput sequencing were consistent with the results obtained from the modified PR-PCR, indicating that the modified PR-PCR has high potential to detect low-frequency mutations (as low as 0.15%). By contrast, the rare rifampin-resistant mutations in these samples (H526Y of sample S748, and S531L of samples S479 and S643) could not be detected by the conventional AS-PCR (S3A and S3B Fig). Even when the DNA template input was increased ten times to 50 ng, these rare mutations still failed to be detected as well (S3C Fig).

### Long-fragment amplification of the modified PR-PCR

To evaluate its applicability in long-fragment amplification, we used the modified PR-PCR to amplify 1 kb and 1.2 kb fragments using gDNA from cell lines and the clinical samples, respectively, and compared the sensitivities with those in the amplification of 355 bp and 322 bp fragments, respectively. The results showed that the modified PR-PCR can specifically and efficiently amplify long fragments (1–1.2 kb) as well as short fragments (322–355 bp) (S4A Fig). The sensitivities in long and short fragment amplification appeared to be consistent (S4B Fig and Fig 5C). These indicate that the modified PR-PCR can be used in long-fragment amplification as well.

In conclusion, we developed a novel modified PR-PCR method that uses a ddNTP-blocked primer and an enzyme combination of a routine amount of Taq DNA polymerase and a tiny amount of high-fidelity DNA polymerase. This method can be used for easy detection of various mutation types, including point mutations, insertions and deletions, with high selectivity and sensitivity. It is also suitable for long-fragment amplification. In addition, the method can be further developed into a closed-tube reaction by adding fluorescent dye (e.g. SYBR Green I, SYTO green-fluorescent nucleic acid stains) or using a probe in the future. Currently, the 3'-ddC-blocked primer can be commercially synthesized and other kinds of dideoxynucleotide-blocked primers can be obtained by adding a specific dideoxynucleotide to the 3'-end of an oligodeoxynucleotide via terminal transferase [14,28]. Therefore, the modified PR-PCR represents a simple, sensitive and promising approach in mutation detection, molecular diagnosis and other relevant areas.

## Supporting Information

S1 Fig Detection of point mutation, insertion and deletion using the conventional AS-PCR and the typical PR-PCR. (a) The conventional AS-PCR mediated by Taq DNA polymerase. (b) The typical PR-PCR method mediated by high fidelity DNA polymerase and ddC-blocked primer. All PCR assays were performed in a total volume of 20 μL containing various amount (0.5, 1, 1.5 and 2 U) of Taq or PrimeSTAR HS DNA polymerase and 1 μL of plasmid template at a concentration of 10^7^ copies/μL. The cycling conditions were pre-denaturation at 94°C for 2 min, followed by 30 cycles of denaturation at 94°C for 20 s, annealing at 56°C for 20 s and extension at 72°C for 25 s. NTC: no-template control.
(TIF)

S2 Fig Selectivity and sensitivity of the modified PR-PCR in detection of mutant plasmids. (a) The modified PR-PCR method. (b) The modified PR-PCR method using fusion blocked primer coupled with an adaptor. In the sensitivity experiment, the template input of mutant plasmids were at a concentration ranging from 5 × 10^7^ copies/μL to 50 copies/μL and the wild-type plasmid template was at a concentration of 5 × 10^7^ copies/μL. In the selectivity experiment, a series of mutant type plasmid templates and a series of mixture templates containing 10%, 1%, 0.5%, 0.1%, 0.05%, 0.01%, 0.005% and 0.001% mutant plasmids among wild-type plasmids at a concentration of 5 × 10^7^ copies/μL were used. The modified PR-PCR assays were performed under the conditions described in Fig 4. (C) Melting curve analysis of amplicons produced via the modified PR-PCR. The modified PR-PCR could detect a frequency of 5 × 10^−5^ mutant alleles among wild-type DNA when the fusion-blocked primer and adaptor were used.
(TIF)

S3 Fig Detection of mutations in rifampin-resistant *Mycobacterium tuberculosis* using the conventional AS-PCR. (a) Primer and template sequences. WT-F and Mu-F indicate the diagnostic primers that completely match the wild-type and mutant alleles, respectively. For other details, please see Fig 7. (B) Gel electrophoresis of the amplicons obtained using the AS-PCR with 5 ng of genomic DNA. All reactions were performed in a total volume of 20 μL containing a mixture of 0.5 U of Taq DNA polymerase, 0.3 μM each of forward and reverse primer, 0.2 mM dNTPs, (1×) PCR Buffer and 5 ng (equal to approximately 1×10^6^ copies) of template. The PCR cycling condition was pre-denaturation at 98°C for 2 min, followed by 35 cycles of denaturation at 98°C for 10 s, annealing at 55°C for 15 s and extension at 72°C for 15 s. The positive (TB-526PC and TB-531PC) and negative control plasmids were both amplified, regardless of whether WT-F or Mu-F was used. The mutants present at a low frequency could not be amplified using the mutant primer (H526Y for sample S748, and S531L for samples S479 and S643). (c) Gel electrophoresis of the amplicons by the AS-PCR with 50 ng of genomic DNA (equal to approximately 10^7^ copies). For other details, please see panel (b). The samples carrying low-frequency mutants are highlighted by asterisks above the names.
(TIF)

S4 Fig Comparison in amplification of fragments with different sizes by the modified PR-PCR. (a) Amplification of 1 kb and 355 bp fragments from gDNA of cell lines, and 1.2 kb and 322 bp fragments from gDNA of clinical samples. The templates input were at a concentration of 20 ng/μL. Both the 1 kb and 355 bp fragments were used to detect *TP53* germ-line mutation R306X in cell line HCC1937, and both the 1.2 kb and 322 bp fragments were used to detected *TP53* germ-line mutation P72R in clinical sample S518. Cell line MCF-7 and clinical sample S521 were used as the wild-type controls. (b) Sensitivity comparison in amplification of 355 bp and 1 kb fragments from gDNA of cell lines, and that of 322 bp and 1.2 kb fragments from gDNA of clinical samples. All assays were performed as described in Fig 5. Sequences of all primers used in the assays are shown in S2 Table.
(TIF)

S1 Table Comparison of main technologies for mutation detection. (DOC)

S2 Table Sequences of the primers used for detection of germ-line mutations R306X and P72R in the *TP53* gene. (DOC)
