# Supplementary figures and images for: Modified Proofreading PCR for Detection of Point Mutations, Insertions and Deletions Using a ddNTP-Blocked Primer
Source: PLoS One. 2015 Apr 27;10(4):e0123468. doi: 10.1371/journal.pone.0123468 (PMC4411138; doi:10.1371/journal.pone.0123468)

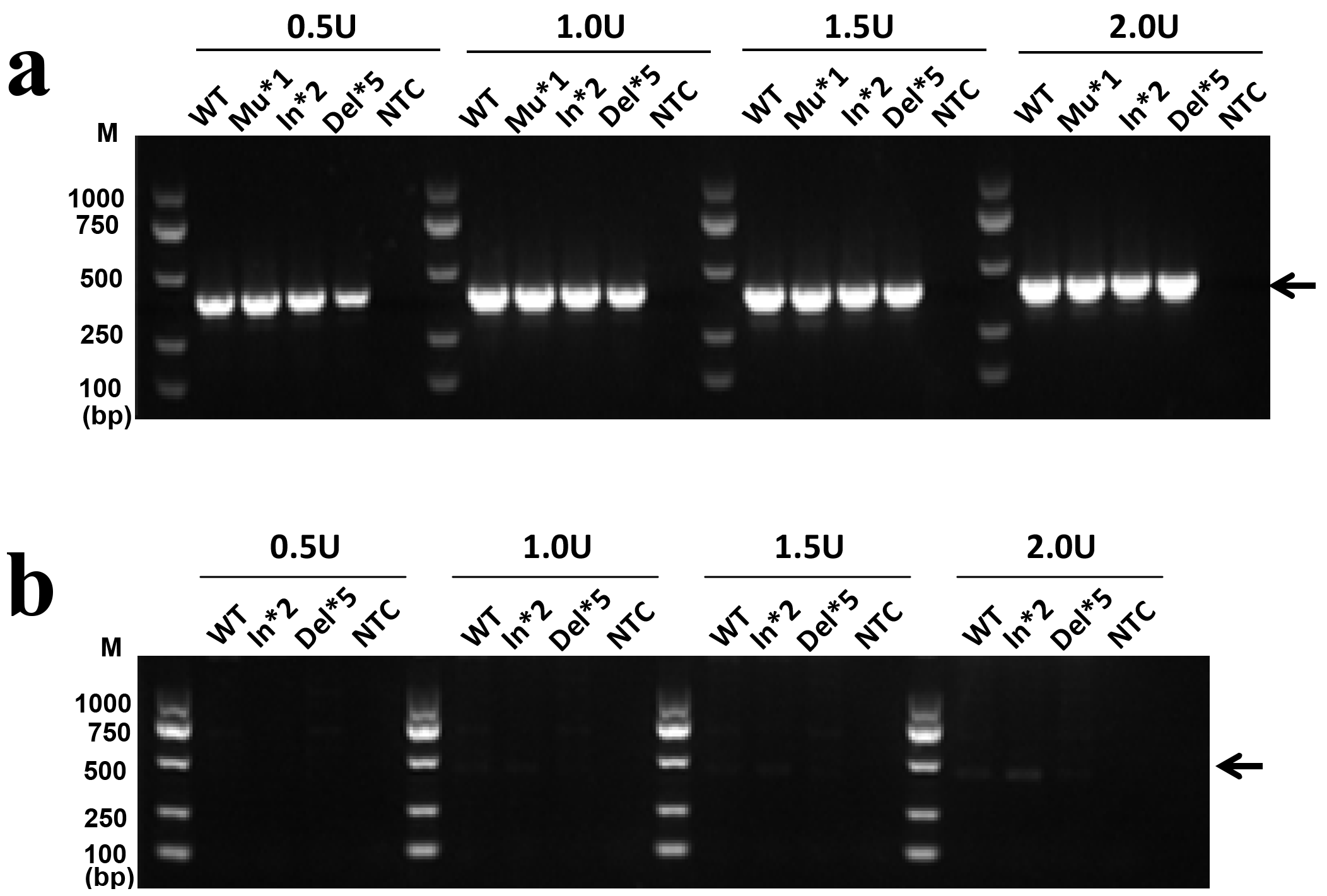

Supplement: S1 Fig — (a) The conventional AS-PCR mediated by Taq DNA polymerase. (b) The typical PR-PCR method mediated by high fidelity DNA polymerase and ddC-blocked primer. All PCR assays were performed in a total volume of 20 μL containing various amount (0.5, 1, 1.5 and 2 U) of Taq or PrimeSTAR HS DNA polymerase and 1 μL of plasmid template at a concentration of 107 copies/μL. The cycling conditions were pre-denaturation at 94°C for 2 min, followed by 30 cycles of denaturation at 94°C for 20 s, annealing at 56°C for 20 s and extension at 72°C for 25 s. NTC: no-template control. (TIF) [file pone.0123468.s001.TIF]

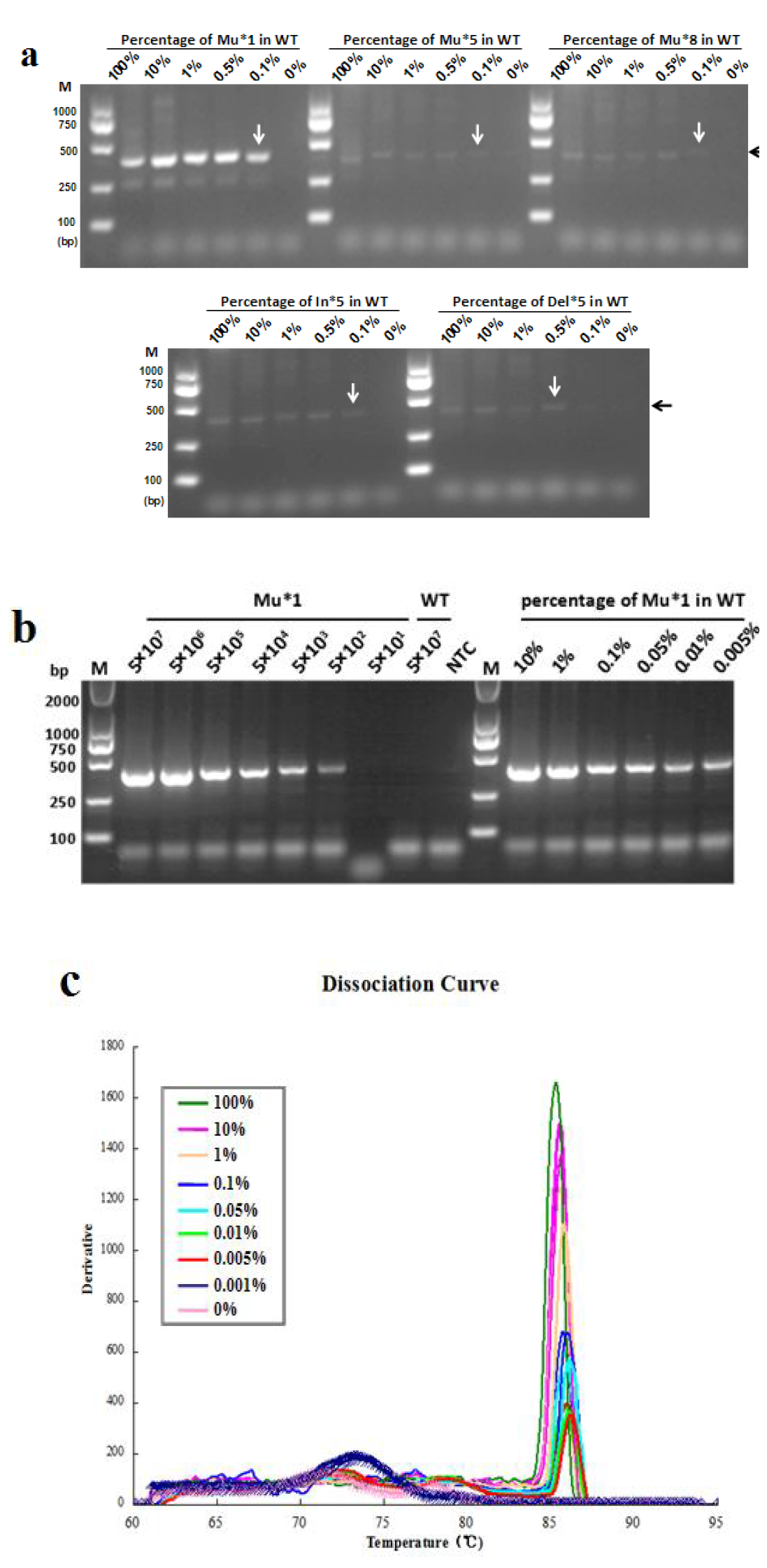

Supplement: S2 Fig — (a) The modified PR-PCR method. (b) The modified PR-PCR method using fusion blocked primer coupled with an adaptor. In the sensitivity experiment, the template input of mutant plasmids were at a concentration ranging from 5 × 10^7^ copies/μL to 50 copies/μL and the wild-type plasmid template was at a concentration of 5 × 10^7^ copies/μL. In the selectivity experiment, a series of mutant type plasmid templates and a series of mixture templates containing 10%, 1%, 0.5%, 0.1%, 0.05%, 0.01%, 0.005% and 0.001% mutant plasmids among wild-type plasmids at a concentration of 5 × 10^7^ copies/μL were used. The modified PR-PCR assays were performed under the conditions described in Fig 4. (C) Melting curve analysis of amplicons produced via the modified PR-PCR. The modified PR-PCR could detect a frequency of 5 × 10^−5^ mutant alleles among wild-type DNA when the fusion-blocked primer and adaptor were used. (TIF) [file pone.0123468.s002.TIF]

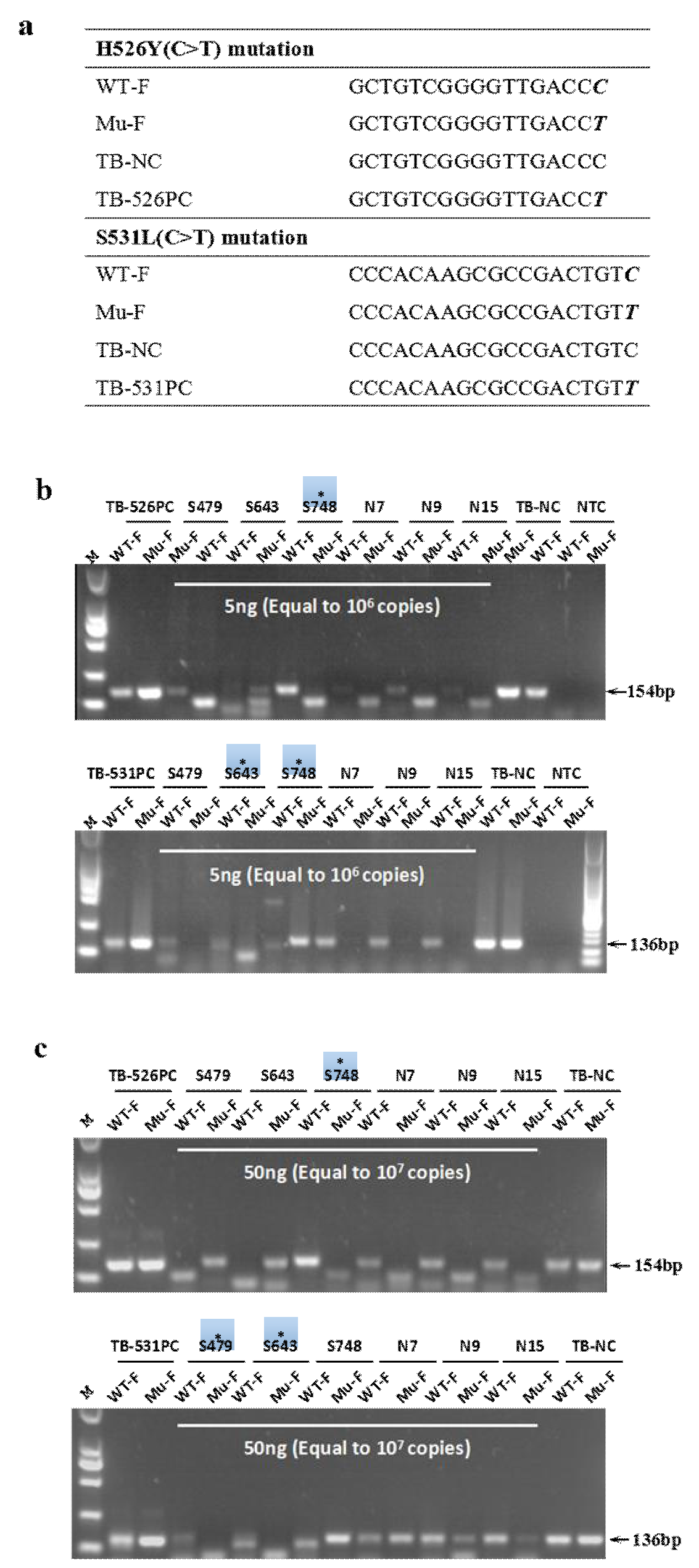

Supplement: S3 Fig — (a) Primer and template sequences. WT-F and Mu-F indicate the diagnostic primers that completely match the wild-type and mutant alleles, respectively. For other details, please see Fig 7. (B) Gel electrophoresis of the amplicons obtained using the AS-PCR with 5 ng of genomic DNA. All reactions were performed in a total volume of 20 μL containing a mixture of 0.5 U of Taq DNA polymerase, 0.3 μM each of forward and reverse primer, 0.2 mM dNTPs, (1×) PCR Buffer and 5 ng (equal to approximately 1×10^6^ copies) of template. The PCR cycling condition was pre-denaturation at 98°C for 2 min, followed by 35 cycles of denaturation at 98°C for 10 s, annealing at 55°C for 15 s and extension at 72°C for 15 s. The positive (TB-526PC and TB-531PC) and negative control plasmids were both amplified, regardless of whether WT-F or Mu-F was used. The mutants present at a low frequency could not be amplified using the mutant primer (H526Y for sample S748, and S531L for samples S479 and S643). (c) Gel electrophoresis of the amplicons by the AS-PCR with 50 ng of genomic DNA (equal to approximately 107 copies). For other details, please see panel (b). The samples carrying low-frequency mutants are highlighted by asterisks above the names. (TIF) [file pone.0123468.s003.TIF]

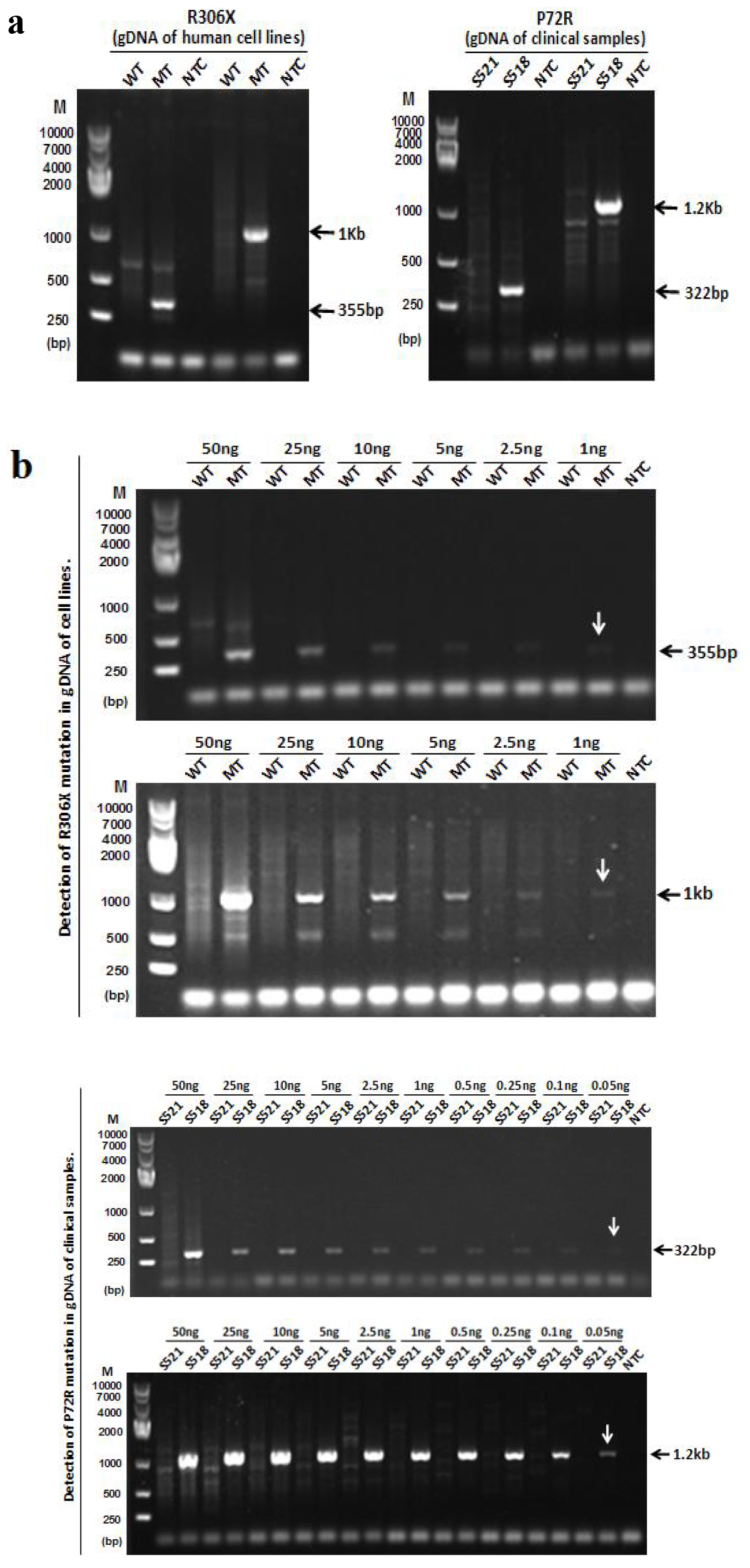

Supplement: S4 Fig — (a) Amplification of 1 kb and 355 bp fragments from gDNA of cell lines, and 1.2 kb and 322 bp fragments from gDNA of clinical samples. The templates input were at a concentration of 20 ng/μL. Both the 1 kb and 355 bp fragments were used to detect TP53 germ-line mutation R306X in cell line HCC1937, and both the 1.2 kb and 322 bp fragments were used to detected TP53 germ-line mutation P72R in clinical sample S518. Cell line MCF-7 and clinical sample S521 were used as the wild-type controls. (b) Sensitivity comparison in amplification of 355 bp and 1 kb fragments from gDNA of cell lines, and that of 322 bp and 1.2 kb fragments from gDNA of clinical samples. All assays were performed as described in Fig 5. Sequences of all primers used in the assays are shown in S2 Table. (TIF) [file pone.0123468.s004.TIF]
