# Supplementary material for: Modified Proofreading PCR for Detection of Point Mutations, Insertions and Deletions Using a ddNTP-Blocked Primer
Source: PLoS One. 2015 Apr 27;10(4):e0123468. doi: 10.1371/journal.pone.0123468 (PMC4411138; doi:10.1371/journal.pone.0123468)
Supplement: S1 Table — (DOC) [file pone.0123468.s005.doc]

**Supporting Information**

**S1 Table. Comparison of main technologies for mutation detection.**

| **Category** | **Method** | **Possibility of false-positive result** | **Selectivity*** | **Rare mutation detection** | **Reference** |
| --- | --- | --- | --- | --- | --- |
| **Allele discrimination based strategies** | ARMS/AS-PCR | High | 10^-1^ – 10^-3^ | No | 3, 22, 23 |
|  | Ligase-mediated amplification reaction(LDR) | High | 10^-1^ – 10^-3^ | No | 6,7 |
|  | PCR-RFLP | Low | 10^-3^ – 10^-4^ | No | 8-10 |
|  | SSCP/DGGE | Low | 10^-1^ – 10^-2^ | No | 11, 12, 32, 33 |
|  | PAP/Bi-PAP | Low | 10^-4^ – 10^-9^ | Yes | 13-15 |
|  | High resolution melting analysis(HRM) | Low | 10^-1^ – 10^-3^ | Yes | 16, 17 |
|  | LNA/PNA-mediated PCR | Low | 10^-2^ – 10^-5^ | Yes | 34-37 |
|  | Proof-reading PCR(PR-PCR) | High | NA | No | 24 |
|  | Modified PR-PCR | Low | 10^-2^ – 10^-5^ | Yes | This study |
| **Sequencing** | Sanger Sequencing | Low | 10^-1^ | No | 18-20 |
|  | Next Generation Sequencing | Low | 10^-2^ – 10^-5^ | Yes | 20, 21 |

***** Data were retrieved directly from the references. NA: not available.

**Supplementary references:**

1. Fodde R, Losekoot M. Mutation detection by denaturing gradient gel electrophoresis (DGGE). Hum Mutat. 1994;3: 83-94.
2. Cariello NF, Scott JK, Kat AG, Thilly WG, Keohavong P. Resolution of a missense mutant in human genomic DNA by denaturing gradient gel electrophoresis and direct sequencing using in vitro DNA amplification: HPRT Munich. Am J Hum Genet. 1988;42: 726.
3. Nielsen PE, Egholm M, Berg RH, Buchardt O. Sequence-selective recognition of DNA by strand displacement with a thymine-substituted polyamide. Science. 1991;254: 1497-1500.
4. Dominguez PL, Kolodney MS. Wild-type blocking polymerase chain reaction for detection of single nucleotide minority mutations from clinical specimens. Oncogene. 2005;24: 6830-6834.
5. Oldenburg RP, Liu MS, Kolodney MS. Selective amplification of rare mutations using locked nucleic acid oligonucleotides that competitively inhibit primer binding to wild-type DNA. J Invest Dermatol. 2007;128: 398-402.
6. Däbritz J, Hänfler J, Preston R, Stieler J, Oettle H. Detection of Ki-ras mutations in tissue and plasma samples of patients with pancreatic cancer using PNA-mediated PCR clamping and hybridisation probes. Br J Cancer. 2005;92: 405-412.
