# Supplementary material for: Modified Proofreading PCR for Detection of Point Mutations, Insertions and Deletions Using a ddNTP-Blocked Primer
Source: PLoS One. 2015 Apr 27;10(4):e0123468. doi: 10.1371/journal.pone.0123468 (PMC4411138; doi:10.1371/journal.pone.0123468)
Supplement: S2 Table — (DOC) [file pone.0123468.s006.doc]

**Supporting Information**

**S2 Table. Sequences of the primers used for detection of germ-line mutations R306X and P72R in the *TP53* gene**.

| **Name** | **Sequence （5′→3′）** | **Tm (°C)** | **Size of amplicon** |
| --- | --- | --- | --- |
| **R306X（c.916C>T） mutation** |  |  |  |
| Wild-type gDNA sequence | TGCCCCCAGGGAGCACTAAG**C** |  |  |
| Mutant-type gDNA sequence | TGCCCCCAGGGAGCACTAAG**T** |  |  |
| 3′-blocked forward primer | TGCCCCCAGGGAGCACTAAG-***ddC*** | 63.4 |  |
| Fusion-blocked forward primer | GACCACCGAAAGTATCGAGTTCATGTCTGCCCCCAGGGAGCACTAAG-***ddC*** | 63.4 |  |
| Adaptor | GACCACCGAAAGTATCGAGTTCATGTC | 61.1 |  |
| Reverse primer 1 | GGAGCCATTGTCTTTGAGGCATCACTG | 62.6 | 355 bp |
| Reverse primer 2 | CTAGCTACTGGGGAGGCAGAGTT | 61.3 | 1 kb |
| **P72R（c.215C>G） mutation** |  |  |  |
| Wild-type gDNA sequence | AATGCCAGAGGCTGCTCCCC**C** |  |  |
| Mutant-type gDNA sequence | AATGCCAGAGGCTGCTCCCC**G** |  |  |
| Fusion-blocked forward primer | CCTCGTTCTACTTGACCTCCATCTTACTAATGCCAGAGGCTGCTCCCC-***ddC*** | 63.4 |  |
| Adaptor | CCTCGTTCTACTTGACCTCCATCTTACT | 61.1 |  |
| Reverse primer 1 | TGACAGGAAGCCAAAGGGTGAAGAG | 61.2 | 322 bp |
| Reverse primer 2 | GGCAGTGCCTCACAACCTCCGT | 63.3 | 1.2 kb |
| **Sequencing primer** |  |  |  |
| Forward primer | GACCTGGTCCTCTGACTGCTCT | 61.4 |  |
| Reverse primer | TGACAGGAAGCCAAAGGGTGAAGAG | 61.2 |  |

Bold font with underline indicates the germ-line mutations R306X and P72R.
